# Pharmacist input to depression screening and management in patients with diabetes: a systematic review

**DOI:** 10.1007/s11096-025-02056-1

**Published:** 2025-11-28

**Authors:** Mai I. AL-Hawamdeh, Fatma Al Raisi, Rana Moustafa Al-Aladawi, Rana Abu-Huwaij, Antonella Pia Tonna

**Affiliations:** 1 University of Jordan, Amman, 11942 Jordan; 2 Department of Pharmacy, School of Pharmacy, Amman Arab University, Amman, 11953 Jordan; 3 Pharmacy Program, Oman College of Health Sciences, Muscat, Oman; 4 Robert Gordon University, Aberdeen, UK; 5 Hamad General Hospital, Hamad Medical Corporation, Doha, Qatar; 6 College of Pharmacy, Qatar University, Doha, Qatar; 7 Department of Pharmacy, Faculty of Pharmacy, Amman Arab University, Amman, 11953 Jordan; 8 School of Pharmacy, Applied Sciences and Public Health, Garthdee Road, Aberdeen, Scotland, UK

**Keywords:** Depression, Diabetes mellitus, Pharmaceutical care, Pharmacists

## Abstract

**Introduction:**

Depression affects approximately 5% of the global adult population, and its clinical and economic burden is particularly pronounced among individuals with chronic conditions such as diabetes mellitus, where it is frequently underdiagnosed and inadequately managed. Pharmacists play an important role in managing chronic diseases, including depression, through screening and medication management.

**Aim:**

This systematic review aimed to critically appraise and synthesize evidence on pharmacist input in screening and managing depression among patients with diabetes, either as sole providers or as part of a multidisciplinary team, and assess their impact on clinical outcomes and quality of life.

**Method:**

A systematic literature search was conducted in March 2023 and updated in March 2025 across Medline, Cumulative Index to Nursing and Allied Health Literature (CINAHL), and International Pharmaceutical Abstracts (IPA) databases, and reported in accordance with the PRISMA 2020 guidelines. Studies, irrespective of their design and published in English, reporting any pharmacist input in screening and managing depression among patients with diabetes, were included. No date restrictions were applied. Data extraction was based on Donabedian’s quality-of-care model, which categorizes findings into structure, process, and outcome. The results were presented in both narrative and tabular formats. Quality was assessed by two independent researchers via the Mixed Methods Appraisal Tool.

**Results:**

Among the 1,852 records screened, 10 studies met the inclusion criteria. The studies described pharmacist input in depression screening n = 4, medication therapy management n = 6, telehealth services n = 2, and shared medical appointments n = 1. The most reported setting was outpatient clinics, n = 5. All studies demonstrated the positive impact of pharmacists on depression screening, treatment initiation, and medication adherence., Some interventions failed to significantly improve clinical parameters such as HbA1c levels or depressive symptoms. Barriers included limited physicians’ response to screening results and a lack of structured care models.

**Conclusion:**

The positive impact of pharmacist interventions on this patient cohort is evident despite the variability across settings and intervention designs, reflecting the adaptability of pharmacist services. Results also suggest the need for standardized models of care and consistent outcome reporting.

**Supplementary Information:**

The online version contains supplementary material available at 10.1007/s11096-025-02056-1.

## Impact statements

- Pharmacist-led interventions in mental health among patients with diabetes have been implemented across various settings, including community pharmacies and specialized clinics, either independently or in collaboration with multidisciplinary teams.
- Pharmacist-led interventions improved depression screening, treatment adherence, and early symptom detection in patients with diabetes.
- The evidence supports the emerging role of pharmacists in addressing mental health needs in diabetes care.
- Clinical outcomes were inconsistent, with physician engagement identified as a key barrier.
- Results suggest that integrated and standardized care models are needed to optimize collaboration and improve both mental health and glycaemic outcomes.

## Introduction

Depression is a prevalent mental health disorder affecting approximately 5% of the global adult population and is associated with decreased quality of life, deterioration of overall health, and increased morbidity and mortality [1, 2]. In 2020, the global economic burden of depression and anxiety was estimated at nearly 1 trillion United States dollars (USD) annually, largely due to productivity loss and healthcare expenditures [3]. The relationship between depression and chronic illness has been increasingly recognized [4–6]. In the United States (US), the prevalence of depression among patients with chronic conditions such as cancer, cardiovascular disease, respiratory disorders, and diabetes mellitus (DM) ranges from 40 to 80%, with adverse effects reported on quality of life (QOL) and health outcomes [6].

It is estimated that one in every six patients with diabetes suffers from depression, with the prevalence doubling among patients with diabetes compared with patients without diabetes [7]. A meta-analysis revealed that depression increases mortality among patients with diabetes by 1.5 times [8]. This growing body of evidence highlights the urgent need to improve the recognition and management of comorbid depression in diabetes care.

Pharmacists are an integral part of the healthcare team and have demonstrated competency in managing chronic diseases, including DM, hypertension, metabolic syndrome, and depression [9–11]. The pharmacist's role in depression management has developed significantly over the years. A meta-analysis of 12 studies investigated the impact of pharmacist intervention among patients with depression. Pharmacists' roles have had a significant impact within different health care settings, with roles in patient education and medication review, and improving patients' medication adherence among patients with depression, among others [12]. Consequently, interest in investigating the potential contribution of pharmacists to the management of depression among patients with diabetes is increasing, given their unique accessibility, expertise in medication management, and potential to deliver both clinical and psychosocial support [13].

### Aim

This systematic review aimed to critically appraise, synthesize, and present the available evidence on pharmacists’ input in the screening and management of depression among patients with diabetes, either as sole providers or as part of a multidisciplinary team. The specific objectives were to (1) identify the settings and models of pharmacist-led interventions and (2) describe the impact of these interventions on patients’ adherence to medications and clinical outcomes, including depression and diabetes-related outcomes.

## Method

### Protocol registration

The protocol for this systematic review was registered with the International Prospective Register of Systematic Reviews (PROSPERO) in May 2023 under the identifier CRD42023413931. The protocol was developed according to the PRISMA-P 2020 (Preferred Reporting Items for Systematic Review and Meta-Analysis Protocols) standards and executed and reported following the PRISMA 2020 guidelines [14].

### Search strategy and information sources

An electronic search across Medline, International Pharmaceutical Abstracts (IPAs), and the Cumulative Index to Nursing and Allied Health Literature (CINAHL) was conducted in March 2023 and updated in March 2025 with no time limits set. The search strategy employed Medical Subject Headings and relevant keywords. Preliminary scoping searches were performed to refine the search strategy. The reference lists of the included studies were manually searched to identify further relevant articles. A comprehensive search strategy was developed and applied across all databases. The detailed search strings used for each database, along with the number of records retrieved, are provided in the Supplementary Material.

All titles and abstracts were independently screened and assessed for inclusion by two reviewers (FA, MA, or RA), and any disagreements were resolved through discussion or referral to a third reviewer. A full text review of papers included followed using the same approach.

### Inclusion and exclusion criteria

The standard systematic review PICO (population, intervention, comparator, and outcomes) protocol was employed.

*Types of population*: Patients with pre-DM, type 1 or type DM for screening with depression for management interventions.

*Types of intervention*: Any pharmacist input (either as sole providers or as part of a multidisciplinary team), in any healthcare settings (inpatient, outpatient, community, and specialized clinics), aimed at improving mental health outcomes in patients already diagnosed with diabetes.

*Types of comparators:* All studies with or without comparators were included.

*Types of outcomes:* All studies evaluating the impact of pharmacist-led interventions on the screening and management of depression among patients with diabetes such as improvements in depressive symptoms, adherence to antidepressant and diabetes medications, and overall diabetes management.

*Types of studies included:* All studies published in English-language peer-reviewed journals, irrespective of study design, without any time restriction, were included.

*Exclusion criteria:* The exclusion criteria included studies not addressing the topic, articles based solely on conceptual models or theoretical frameworks without empirical data (i.e., primary research involving systematically collected and measured data and reported outcomes), gray literature (conference proceedings, abstracts, unpublished studies, commentaries), and narrative or systematic reviews.

### Quality assessment

The quality of the included studies was assessed using the 2018 version of the Mixed Methods Appraisal Tool (MMAT) [20] by two reviewers independently (RA, MA). Any discrepancies were resolved through discussion, and when a consensus could not be reached, a third reviewer (FA) provided input and guided the final decision. The MMAT includes two general screening questions applicable to all study types, followed by specific questions for qualitative studies, randomized controlled trials, nonrandomized trials, quantitative descriptive studies, and mixed-methods studies [15]. Although formal interrater reliability statistics (e.g., Cohen’s kappa) were not calculated, the independent dual-review process with adjudication ensured methodological rigour and minimized bias.

### Data extraction

A customized data extraction was developed to capture the key study characteristics, including publication year, study aim, design, duration, setting (country or institution), participant details (inclusion/exclusion criteria, demographics), pharmacist inputs, key findings or main outcomes, and authors' conclusions. Before full data collection, the data extraction tool was piloted independently by two reviewers (FA, MA) on a subset of included studies to ensure clarity, consistency, and completeness. Minor adjustments were made, and the finalized tool was then applied to all included studies. Numerous conceptual models have been developed to evaluate healthcare quality, with the Donabedian model serving as a foundational framework centered on the triad of structure, process, and outcome [16]. The Donabedian model was used in this SR since it provides a comprehensive and well-established basis for evaluating healthcare quality and has been used successfully in the literature [17–19].

For this systematic review (SR):

The structure considers the resources required for pharmacists to provide care to patients with mental health issues related to diabetes, such as settings, models of care, and the availability of collaborative practice agreements that regulate pharmacists' roles. The process considers interventions performed by pharmacists, including reviewing clinical rounds, involvement in patient management, medication reviews, therapeutic recommendations, pharmacist prescribing, patient counselling, and monitoring. The outcome considers reported outcomes, including clinical parameters, medication adherence, and quality of life. Pharmacist interventions were defined as actions aimed at improving drug use processes as part of patients' or healthcare practitioners’ activities.

### Data synthesis

Owing to the heterogeneity of the data from the included studies (varying patient types, study designs, quality, and outcomes), a meta-analysis was not feasible. Therefore, a descriptive and narrative synthesis was conducted. To ensure robustness in the review process, two independent reviewers entered the data, which was subsequently checked for consistency.

## Results

### Study selection

**Fig. 1:**
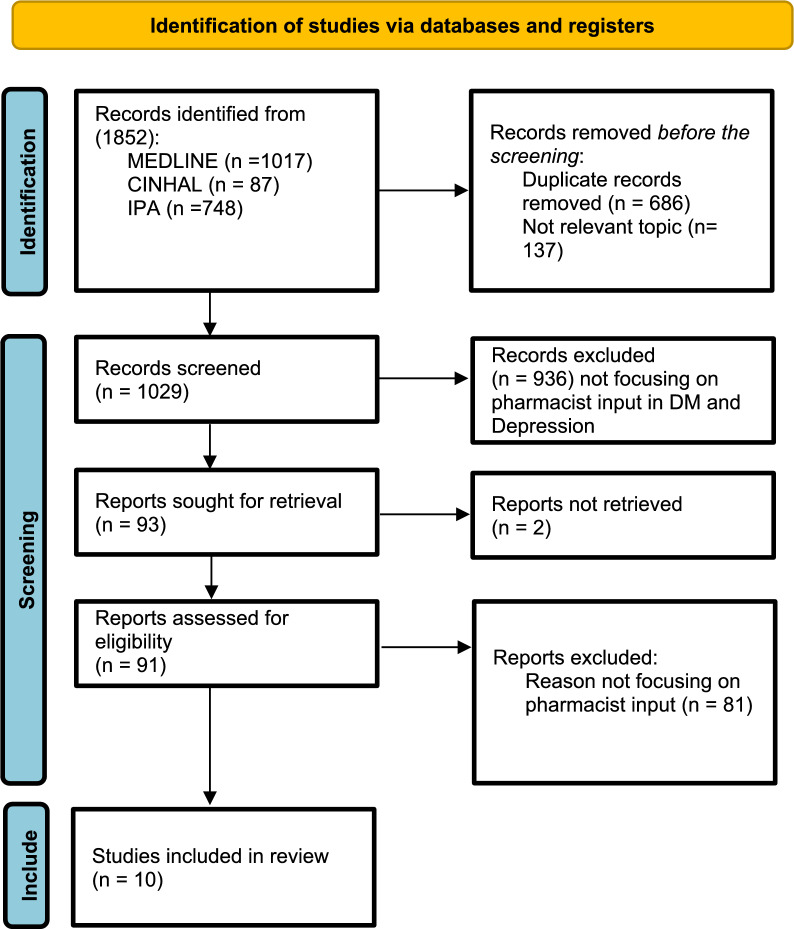
PRISMA 2020 flow diagram for new systematic reviews that included searches of databases and registers only

The systematic review identified 1852 records in MEDLINE, CINAHL, and IPA. Duplicate (n = 686) and nonrelevant records (n = 137) were manually removed, and 1029 records remained for screening. Additional screening of titles and abstracts further excluded 936 records, which did not focus on pharmacist input in depression connected to DM. Ninety-one records remained for full-text retrieval. Ten reports met the inclusion criteria and were included in the final systematic review (Fig. 1). No additional studies were identified through reference list or bibliography searches.

### Quality assessment

The results of the quality assessment of all included studies (n = 10) with the MMAT tool are reported in Tables 1 and 2. The study designs were as follows: randomized controlled trials (RCTs) (n = 3) [23, 24, 31], ad hoc RCTs (n = 1) [25], retrospective cohort studies (n = 2) [28, 30], prospective cohort studies (n = 2) [27, 29], or cross-sectional studies (n = 2) [21, 26]. The quantitative descriptive studies (n = 6 of 10) were of optimal quality, particularly in terms of the research question, sampling strategies, measurement methodology, and response rate; however, a minor limitation was identified in one study with a small sample size, thus limiting the generalizability of the study [20]. (See Table 1). In the case of the RCTs, the quality assessment revealed limitations in the allocation concealment criteria in half of the studies. (Table 2) [21–23].

**Table 1:** Quality assessment tool for quantitative descriptive cross-sectional studies (MMATs)

Quality criteria	Knight DE et al. 2008 (US) [21]	Ragland D et al. 2010 (US) [26]	Rosser S, et al. 2012 (US) [27]	Nazarian PK et al. 2013 (US) [28]	Wilson C, et al. 2018 (US) [29]	Bingham J et al. 2020 (US) [30]
Are there clear qualitative and quantitative research questions (or objectives*), or a clear mixed methods question (or objective*)?	Yes	Yes	Yes	Yes	Yes	Yes
Do the collected data allow address the research question (objective)? E.g., consider whether the follow-up period is long enough for the outcome to occur (for longitudinal studies or study components)	Yes	Can’t tell	Yes	No	Yes	Yes
Is the sampling strategy relevant to address the quantitative research question (quantitative aspect of the mixed methods question)?	Yes	Yes	Yes	Yes	Yes	Yes
Is the sample representative of the population understudy?	Can’t tell	Yes	Yes	No	Yes	Yes
Are measurements appropriate (clear origin, or validity known, or standard instrument)?	Yes	Yes	Yes	Yes	Yes	Yes
Is there an acceptable response rate (60% or above)?	Yes	Yes	Can’t tell	Can’t tell	Yes	Yes

Keywords: Yes: criteria met; No: criteria not met; Can’t tell: not enough information provided to make a decision; and NA: not applicable

**Table 2:** Quality assessment tool for quantitative randomized controlled trials (MMATs)

	Polomoff CM et al. 2021. US [23]	Taveira TH et al. 2021. US [24]	Cohen LB et al. 2020. US [31]	Bateman MT et al. 2023. US [25]
1. Are there clear quantitative research questions or objectives?	Yes	Yes	Yes	Yes
2. Does the collected data address the research question or objectives?	Yes	Yes	Yes	Yes
3. Is there a clear description of the randomization?	No	Can’t tell	Yes	Yes
4. Is there a clear description of the allocation concealment (or blinding when applicable)?	No	No	Yes	No
5. Are there complete outcome data (80% or above)?	No	Yes	Yes	Yes
6. Is there low withdrawal/drop-out (below 20%)?	No	Yes	Yes	Yes

Keywords: Yes: criteria met; No: criteria not met; Can’t tell: not enough information provided to make a decision; and NA: not applicable

### Data extraction

All reviewed reports (n = 10) were conducted in the United States (US). The participants included patients with diabetes mellitus (type 1 or type 2) or prediabetes with depression across various healthcare settings, such as community pharmacies [21, 24, 26], telehealth and outpatient clinics [20, 22, 25–27], Interventions were implemented either solely by pharmacists, by pharmacy students under the supervision of a staff pharmacist, or in collaboration with other healthcare providers. The sample size ranged from small (n = 15) [26] to large (n = 8,167) [27]; the duration of the studies ranged from a few months (n = 3 months) [25] to several years (n = 2.8 years) [24]. Of the 10 included studies, five focused exclusively on patients with type 2 diabetes [26–29, 32], three included both type 1 and type 2 diabetes [25, 30, 34], and two did not specify the diabetes type [31, 33]. Table 3 presents the results of the studies included in this systematic review, describing the role of pharmacists in medication therapy management, screening, and improving the quality of life in diabetic patients with depression, together with the main clinical outcomes.

**Table 3:** Overview of Clinical Characteristics and Main Clinical Outcomes, 2008–2025

Author, study country, and Year	Study design and duration	Study setting	Aim	Participants' population, age & N (at baseline)	Intervention	Control	Main clinical outcomes achieved
Knight DE et al. [20] United States (2008)	Cross-sectional descriptive study (4 months)	An outpatient pharmacist-led clinic in an urban setting, serving an underserved, low-income, inner-city population	To identify possible undiagnosed and undertreated depression in patients with type1 and type 2 DM using screening by a student pharmacist	Adults with type1 and type 2 DM age: ≥ 40 years (N = 45)	The patients completed a 10-min questionnaire, using the Zung Self-rating Depression Scale (SDS). Depression screening is based on an SDS score of 50 or higher. The student pharmacists interviewed the patients and collected the responses, and the screening results were documented the as a clinical note in the patient’s medical chart	No control	Following the intervention, 27% (n = 12 of patients had a current diagnosis of depression. Among diagnosed patients, 25% (n = 3) were adequately treated, 50% (n = 6) undertreated, and 25% (n = 12) untreated. There were no significant differences in the mean (± SD) HbA1C or fasting blood glucose levels between depressed and nondepressed patients
Ragland D et al. [25] United States (2010)	Cross-sectional survey (3 months)	Women’s prenatal outpatient clinic serving local and statewide patients	To determine the prevalence of depression in women with type 1 and type 2 DM receiving prenatal care at a women’s health clinic and assess the appropriateness of depression care received	Pregnant females with type 1 and type 2 DM Mean age 28.7 ± years (6.7) (N = 50)	The clinical pharmacist provided individualized diabetes education and management, while precepting fourth-year pharmacy students. Together, they screened pregnant patients with diabetes for depression using the Beck Depression Inventory-II (BDI-II), documenting scores categorized as minimal (0–13), mild (14–19), moderate (20–28), or severe (≥ 29)	No control	The screening revealed that of the 50 patients included in the study, 42% (n = 21) had clinical depression. Among patients with clinical depression, 19% (n = 4) were receiving current treatment for their condition
Rosser S et al. [28] United States (2012)	Prospective Cohort Study (1 year)	Community pharmacy located within a large grocery chain	To develop, implement, and evaluate a pharmacist-conducted depression screening program in a large chain grocery store pharmacy	Adults visiting a community pharmacy chain as walk-ins Mean age 45 years ± (13.6) (N = 3,726)	Community pharmacists screened patients for depression using the Patient Health Questionnaire-2 (PHQ-2), followed by the PHQ-9 for those who screened positive. Patients with positive PHQ-9 results were referred to their physicians, and pharmacists conducted follow-ups to ensure appropriate management and continuity of care	No control	A total of 3,726 patients were screened, of whom 67 screened positive on the PHQ-2. Among these, 17 also screened positive on the PHQ-9 and met the criteria for depression. These patients were referred to their physicians; five reported suicidal thoughts and were referred for urgent care. At follow-up, 58.8% (10 of 17) had treatment initiated (n = 6) or modified (n = 4)
Nazarian PK et al. [26] United States (2013)	Retrospective chart review (6 months)	Once weekly a collaborative practice psychiatric pharmacist clinic. Patients were referred by their primary care provider or psychologist	To describe the outcome of depression management in patients with diabetes treated by a psychiatric pharmacist within a collaborative practice model	Adults with DM (type was not specified) and depression in a safety-net clinic Average age 55.6 years (N = 15)	The psychiatric pharmacist confirmed major depressive disorder (MDD) using Diagnostic and Statistical Manual of Mental Disorders, Fourth Edition, Text Revision.(DSM-IV-TR) criteria, performed chart reviews, and monitored PHQ-9 scores, HbA1c, and treatment plans. Under a collaborative practice agreement, the pharmacist followed the American Psychological Association (APA) guidelines and was authorized to initiate, adjust, or discontinue medications and order laboratory tests	No control	A response was defined as a reduction of 50% or more in the PHQ-9 score from baseline, while remission was defined as achieving a PHQ-9 score of less than 5 A therapeutic response was observed in 89% of patients (n = 8), while remission was achieved in 33% (n = 3)
Wilson et al. [24] United States (2018)	Prospective cohort (2 years and 8 months)	Community pharmacy chain involving an independent community pharmacy-run diabetes education center	To test the feasibility of implementation and integration of community pharmacist-led depression screening for patients with type 2 DM in an underserved rural area and to assess the response rate of prescribers to pharmacist-led depression screening and treatment recommendations	Adults with type 2 DM Age 65 [50–80] (N = 57)	Community pharmacists administered the PHQ-9 to patients enrolled in disease management programmes, recording baseline HbA1c levels. Patients were categorized based on screening results of PHQ-9 and followed up at 6 months and 1 year to reassess HbA1c, evaluating the effect of depression treatment on glycaemic control	No control group	Of the 64 patients who met the inclusion criteria, 11% (n = 7) declined to take the screening. Of the 57, 11 were positive (19.3%) and 46 were negative (80.7%) for possible depression. None of these providers elected to initiate treatment based on the screening results. As a result, no data were available to evaluate the effects of depression treatment on diabetes outcomes
Bingham J et al. [27] United States (2020)	Retrospective longitudinal review (1 year)	National pharmacist-led tele-health medication therapy management (MTM)	To evaluate the impact of a targeted pharmacist-led tele-health intervention to increase adherence to antidepressant medications amongst patients with type 2 DM	Adults with type 2 DM and mental health conditions Mean age 63 ± 11 (n = 8167)	The pharmacist assessed patients’ adherence to antidepressants using the proportion of days covered (PDC), performed comprehensive medication review, identified potential causes of nonadherence and provided personalized counselling ensuring optimal medication use and patient support	No control	Community pharmacist-led interventions resulted in a significant improvement in adherence to psychotropic medications. The overall mean PDC for all medications in the 6 months before the intervention was 66% ± 12%. In the 6 months after the intervention, the overall mean PDC rose to 79% ± 19%
Cohen LB et al [29] United States (2020)	Open-label randomized controlled (6 months)	Veterans Administration Medical Center	To determine whether a pharmacist-led tele-health disease management programme is superior to the usual care of nurse-led tele-health in improving diabetes medication adherence, HbA1C, and depression scores in patients with concomitant type 1 and type 2 DM and depression	Veterans with type 1 and type 2 DM and depression Case group mean age = 63.1 years ± (12.2). Control group mean age = 60.5 years ± (9.4) (N = 30)	Patients with type 1 or type 2 diabetes and comorbid depression were randomized to either a clinical pharmacist-led tele-health or nurse-led telehealth group. In addition to standard education on self-management of blood glucose and weight, patients in the pharmacist-led group received a comprehensive medication review to optimize therapy, address medication-related issues, and improve management of both conditions. Pharmacists were also authorized to order laboratory tests and prescribe medications as needed	The usual care provided through the nurse-led tele-health programme	At 6 months follow-up, the pharmacist-led tele-health arm showed significant improvements to overall medication adherence (13.9; 95% CI 6.6 to 21.2), including cardiovascular and antidepressant medications. However, there was no improvement in HbA1c and no difference in depression score PHQ-9 compared to the control group
Polomoff CM et al [21] United States (2021)	Randomized Control Trial (15 months)	Community based intervention targeting Cambodian Americans in the U.S. Self-report surveys were collected at baseline, 12 months, and 15 months	The study compared three intervention arms with a control group for changes in self-reported adherence, perceived barriers, and medication beliefs: (1) community health worker–delivered care, (2) lifestyle programme (Eat, Walk, Sleep), and (3) Eat, Walk, Sleep plus pharmacist and CHW-delivered medication therapy management (EWS + MTM)	Cambodian Americans with type 2 DM Age 35–75 (N = 188)	Clinical pharmacists led personal interviews with the participants, facilitated the undertaking of the survey, and led the training of CHWs in MTM. CHWs contributed to the medication therapy management process by conducting medication history-taking and language translation	No	Medication therapy management delivered by a cross-cultural team with a CHW and pharmacist resolved 84% of drug therapy problems. High satisfaction and strong therapeutic alliance reported by the participants
Taveira TH et al. [22] United States (2021)	Randomized Control Trial (5 months)	Shared medical appointments (SMAs) within the Veterans Affairs (VA) healthcare system provided multidisciplinary education and pharmacist-led interventions, involving patients’ family members and social support networks	To test the efficacy of pharmacy-led SMA on HbA1C values and changes in depression symptoms	Older adults with Type 2 DM and depression Age 60 years ± 10 (N = 44)	Clinical pharmacists led pharmacologic and behavioral intervention portions as part of a 2-h, once-weekly sessions for 4 weeks, followed by 5 monthly booster sessions. The pharmacist provided structured group learning with peers, educated the patients to set their therapeutic goals, and about the management of DM, HTN, weight management, and smoking cessation, along with pharmacological intervention for glycemic control. No intervention was done for depression	Standard care arm	Significant improvement in glycemic control in the intervention arm; however, there was no significant change in depressive symptoms for either arm
Bateman MT et al. [23] United States (2023)	A post hoc subgroup analysis of a diabetes-focused RCT (12 months)	This is a post hoc subgroup analysis of a diabetes-focused randomized controlled	To evaluate whether glycaemic control and depressive symptoms improve for patients with diabetes and depression with additional management from clinical pharmacists compared with those receiving the standard of care	Veterans with with type 2 DM and depression Age: Control group mean age 51 years Intervention group mean age 52.1 years (N = 132)	Patients received either usual care from their primary care provider or additional collaborative care involving a clinical pharmacist. The pharmacists conducted medication reviews, supported self-management goal setting, implemented therapy adjustments, and documented follow-up visits. Depression screening using the PHQ-9 was performed at baseline and at 6 months to monitor progress and guide care modifications	Standard care: managed by the primary care provider	The HbA1C improved from baseline to 6 months by − 2.4% % (SD, 2.41) compared to − 0.1% (SD, 1.78) among patients in the control group (*P* 0.0081). The intervention and control groups showed similar improvements in PHQ-9 scores (− 6.11 and − 5.68 points, respectively) (P = 0.41). Pharmacists detected a greater average number of Medication Therapy Problems (MTPs) per patient in the depressed group (11.7% n =) compared to the nondepressed group (7.4%n = , resulting in more focused interventions

### Pharmacist interventions

The reviewed studies provided varied levels of detail regarding the pharmacist settings and recruitment. Two studies were conducted in community pharmacies [24, 28], while two utilized telehealth models to deliver pharmaceutical care [30, 31]. An additional six studies took place within outpatient clinics or primary healthcare centers [21, 23–26, 28]. Within these settings, in five studies, pharmacists were involved in screening for depression in individuals with diabetes or prediabetes [21, 25, 27–29], whereas in the other five studies, pharmacist interventions focused on managing already diagnosed depression among patients with diabetes. [23, 24, 26, 30, 31]. Pharmacy students under pharmacist supervision were involved in delivering interventions in two studies in primary care settings [21, 26], community pharmacists delivered another two [27, 29], a specialist psychiatric pharmacist led one intervention [28], and clinical pharmacists delivered five interventions in outpatient clinics [23–25, 30, 31].

The majority of patients were recruited through convenience sampling methods such as walk-ins [27] or proactive identification methods, such as patient chart reviews or direct invitations based on eligibility criteria [21, 23, 26–31]. In two studies, patients were referred through collaborative practice agreements [24, 25].

### Process (care provided)

Several pharmacists' interventions have been reported, with a primary focus on screening, medication management, and longitudinal follow-up. Depression screening was conducted in four studies via validated instruments: the Patient Health Questionnaire-2 and -9 (PHQ-2/PHQ-9 [27, 29], the Beck Depression Inventory-II (BDI-II) [26], and the Zung Self-Rating Depression Scale [21]. The studies focusing on depression management reported multicomponent interventions featuring medication optimization through comprehensive medication reviews and drug-related problem identification, pharmacist prescribing when dose adjustments were needed [25, 28, 30], and laboratory monitoring [31]. One study reported a lack of physician response, for some screened positive for depression using the PHQ-9, limiting the ability to assess the interventions’ impact [31]. Patient education components focused on therapeutic goal setting, diabetes self-management training, and weight control counselling [24].

### Clinical outcomes

Screening studies revealed a substantial prevalence of undiagnosed depression, with rates ranging from 19.8 to 42% among the studied populations. In most cases, participants are referred to primary care physicians for further evaluation and management [26, 27]. Follow-up audits indicated that a significant proportion of referred patients (60–70%) were initiated on treatment following these referrals [27, 29], whereas other studies reported the communication of screening results to primary providers without further intervention tracking [21, 28].

Six studies evaluated the outcome of pharmacist interventions in managing depression and diabetes in comorbid patients. Of them, two studies reported statistically significant improvements in glycemic control [24, 25], while, study reported measurable reductions in depressive symptoms in 89% of the patients and resolution in one-third of the patients [28]. One study revealed a greater HbA1c reduction in patients with depressive symptoms receiving pharmacist care (2.4 vs. 0.1 percentage points; *P* = 0.0081) but no change in depressive symptoms [25]. Comparative analysis indicated that pharmacist-led care was associated with increased adherence to glycemic and/or antidepressant medications in two studies [30, 31]. Two studies highlighted the role of pharmacists in identifying drug-related problems (DRPs), with tailored interventions resolving 84% of DRPs in one study [23] and an average of 11.7 identified DRPs per patient in another [25]. Notably, one study reported no clinically meaningful changes in glycemic control or depression severity following pharmacist intervention [31].

## Discussion

This systematic review aimed to synthesize the evidence on the pharmacist’s role in screening and managing depression among patients with diabetes and to describe the various practice settings in which these interventions were implemented. Pharmacist-led interventions were conducted across diverse settings, including community pharmacies, outpatient clinics, and telehealth. The outcomes of these interventions varied: depression screening helped identify previously undiagnosed patients, while management interventions improved both diabetes and depression medication adherence, reduced depressive symptoms, and enhanced diabetes control.

The included studies in this review demonstrated both the effectiveness and adaptability of pharmacist-led interventions across a wide range of healthcare settings, including community pharmacies, outpatient clinics, and inpatient services. These findings are consistent with the literature highlighting the positive impact of pharmacist involvement in the care of patients with comorbid chronic conditions, irrespective of setting, and including hypertension, heart failure, metabolic syndrome, and chronic kidney disease [30–33]. This diversity of setting is further supported by findings from other systematic reviews examining the role of pharmacists, specifically in managing depression [34]. One review reported that depression screening predominantly occurred in community pharmacy settings, with 21 out of 26 studies conducted in such environments. In contrast, another systematic review revealed that pharmacist-led management of depression was implemented primarily in inpatient settings, with 14 out of 15 studies conducted in hospitals [35]. This demonstrates that community pharmacy may be the ideal setting for screening, while secondary care interventions are more supportive of symptom management and are indeed similar to findings from our review.

The results of this SR revealed variability in clinical outcomes, including depression scores and glycaemic control, which may be due to multiple factors, including differences in intervention intensity, pharmacist training, the integration of pharmacist intervention within care teams, and the duration of follow-up. It is evident in the literature that improvement in depressive symptoms is associated with better glycemic control. This relationship may be explained by enhanced overall well-being and increased motivation to adhere to diabetes medications, maintain a healthy diet, and adopt positive lifestyle behaviors once depression is effectively managed [36].

A particularly significant finding in this review was the underutilization of pharmacist-led interventions in the form of depression screening, where provider follow-up was either absent or undocumented in many studies. In support of our findings, a systematic review conducted by Keven Oua and colleagues on pharmacist-led mental illness screening identified a similar gap in practice. Among the 26 studies reviewed, only 9 reported how the screening results were communicated to physicians to initiate appropriate treatment [34]. This highlights a critical gap in translating evidence into effective management and reflects systemic challenges such as unclear referral pathways, lack of integration into mental health services, or insufficient policy support.

Overall, while this review highlights the potential value of pharmacists in managing patients with diabetes and depression, the full realization of the pharmacist’s potential impact requires structured implementation strategies, multidisciplinary collaboration, and outcome-driven research. Further research should prioritise well-designed randomized controlled trials with standardized interventions, longer follow-up periods, and the inclusion of economic, social, and humanistic outcomes to better measure the value that pharmacists can bring to this complex patient population.

This review has several limitations that should be considered when interpreting the findings. First, restricting the search to English-language publications may have introduced language bias, although this decision was made to ensure accurate data extraction and appraisal by the reviewing team. Future reviews could make use of reliable AI-driven translation tools to include non-English studies and expand the evidence base. Second, the number of studies meeting the inclusion criteria was limited (n = 10), and all were conducted in the US, which may affect the generalisability of the results to other healthcare settings or populations. Third, the lack of consistent reporting of effect sizes across studies limits the depth of synthesis, highlighting the need for future research to report both p values and effect sizes to improve comparability and interpretation. Finally, differences in study duration and sample size across the included studies may have contributed to heterogeneity in the reported outcomes. Shorter studies may not have captured long-term effects, while smaller sample sizes limited the statistical power and generalizability of the findings.

Finally, pharmacist-led interventions for depression in patients with diabetes are complex and involve multiple interacting components requiring behavioral changes from both patients and providers, which may contribute to variable outcomes. However, none of the included studies fully adhered to the UK Medical Research Council (MRC) framework for complex interventions [37], with notable gaps in the development and feasibility phases, and no references to separate publications reporting these stages. Similarly, prior systematic reviews examining the role of pharmacists in depression among patients with chronic illnesses have demonstrated limited integration of the MRC framework [34, 35]

Despite these limitations, this systematic review has several strengths. The overall quality of the studies included was generally good, supporting the robustness of the findings. This review addresses a critical gap in the literature by providing evidence on the role of clinical pharmacists in depression among patients with diabetes, thereby contributing to the understanding of pharmacist interventions and their impact on this population. Moreover, this highlights the need for future research to apply more robust statistical methodologies when evaluating the clinical outcomes of pharmacist interventions.

## Conclusion

The limited body of research addressing pharmacists' involvement in managing depression among patients with diabetes offers preliminary evidence of positive outcomes from screening and management within collaborative care models. However, the reviewed studies were predominantly conducted in a single country, highlighting the need for further research across diverse geographical regions. This broader scope would enhance the generalizability of the findings. Additionally, further studies are needed to establish stronger evidence regarding both the clinical effectiveness and cost-effectiveness of these interventions.

## Supplementary Information

Below is the link to the electronic supplementary material.

Supplementary file1 (DOCX 17 KB)

## Data Availability

The datasets generated during and/or analysed during the current study are available from the corresponding author upon reasonable request.
