# Correction: Pharmacist input to depression screening and management in patients with diabetes: a systematic review

**DOI:** 10.1007/s11096-025-02075-y

**Published:** 2025-12-20

**Authors:** Mai I. AL-Hawamdeh, Fatma Al Raisi, Rana Moustafa Al-Aladawi, Rana Abu-Huwaij, Antonella Pia Tonna

**Affiliations:** 1 University of Jordan, Amman, 11942 Jordan; 2 Department of Pharmacy, School of Pharmacy, Amman Arab University, Amman, 11953 Jordan; 3 Pharmacy Program, Oman College of Health Sciences, Muscat, Oman; 4 Robert Gordon University, Aberdeen, UK; 5 Hamad General Hospital, Hamad Medical Corporation, Doha, Qatar; 6 College of Pharmacy, Qatar University, Doha, Qatar; 7 Department of Pharmacy, Faculty of Pharmacy, Amman Arab University, Amman, 11953 Jordan; 8 School of Pharmacy, Applied Sciences and Public Health, Garthdee Road, Aberdeen, Scotland, UK

**Correction to: International Journal of Clinical Pharmacy** 10.1007/s11096-025-02056-1

In this article, the author’s name Fatma Al Raisi was incorrectly written as Fatma l Raisi.

The original article has been corrected.

