# Supplementary material for: Pharmacist input to depression screening and management in patients with diabetes: a systematic review
Source: Int J Clin Pharm. 2025 Nov 28;48(2):376–92. doi: 10.1007/s11096-025-02056-1 (PMC12992472; doi:10.1007/s11096-025-02056-1)
Supplement: Supplementary file 1 — Supplementary file1 (DOCX 17 KB) [file 11096_2025_2056_MOESM1_ESM.docx]

**Supplementary material**

**Search Strategy**

Boolean operators such as truncations (*), wildcards ($), and adjacency searches (e.g., adj2) were utilized where applicable. All the search results were imported into Refworks^®^ for reference management and duplicate removal.The following grouped terms were searched independently and then in combination by three reviewers (FA, MA, and RA):

**The** specific search strings **used for all data bases**

Pharmacist-related terms: “pharmacist” [MH+], “pharmacy” [MH+], “Pharma*” [MH], “Pharmac*” [MH], “clinical pharmacist*” [TI/AB/SU], “clinical pharmacy” [TI/AB/SU], “clinical pharmacies” [TI/AB/SU], “community pharmacist*” [TI/AB/SU], “community pharmacy” [TI/AB/SU], “pharmacies” [TI/AB/SU], “pharmacy” [TI/AB/SU] with the OR operator. Mental health terms: “mental health” [MH+], “psychology” [MH+], “depression” [MH+], “mental health” [TI/AB/SU], “psychology” [TI/AB/SU], “depression” [TI/AB/SU] with the OR operator. Diabetes-related terms: “diabetes” [MH+], “diabetic” [MH+], “type 2 DM” [MH+], “diabetes” [TI/AB/SU], “diabetic” [TI/AB/SU] with the OR operator. All related searches were subsequently combined with the AND operator.

**Databases**

The following databases will be searched:

1. PubMed
2. CINAHL
3. International Pharmaceutical Abstracts
